# Epitome of Skin Diseases

**Published:** 1876-12

**Authors:** 


EPITOME OF SKIN DISEASES, WITH FORMULÆ, FOR STUDENTS AND PRACTITIONERS. By Tilbury Fox, M.D.; F. R. C. P., Physician to the Department for Skin Diseases in University College Hospital; Author of various works on Skin Diseases, etc., and T. C. Fox, B. A., (cantab), M. R. C. S. Philadelphia: Henry C. Lea. 1876.

In this little volume of one hundred and twenty pages the author admits freely the intricacy and bewildering tendency of works on skin diseases. He speaks of the progress in the way of elucidating these obscurities by late researches, and himself, in this work, attempts to simplify the classification usually adopted. His ideas as to the pathological affinities and analogies in skin affections are very reasonable. Had he presented or even suggested a nomenclature or classification based upon these views, instead of that based upon the "strange topics or data" of the older writers, the work would have been much more thorough than more voluminous books on this subject. It is, however, a book well worth perusal by all interested in this branch of the profession. As an epitome the leading features in dermatology are succinctly presented with appropriate treatment for the various diseased conditions. Altogether we think it less liable to create confusion than any work of the kind that we have seen.

